# Avulsion Fracture of the Tibial Tuberosity Combined with Lateral Tibial Plateau in an Adolescent

**DOI:** 10.1155/2018/4198379

**Published:** 2018-11-18

**Authors:** Jameel Mahmoud, Bander S. Alrashedan, Khalid M. Allimmia, Basam Alanazi, Talal A. Alshehri

**Affiliations:** Department of Orthopedic Surgery, Ministry of Health (MOH), King Saud Medical City, Ulaishah, 7790 Al Imam Abdul Aziz Ibn Muhammad Ibn Saud, Riyadh 12746 3617, Riyadh 12746, Saudi Arabia

## Abstract

Avulsion fractures of the tibial tuberosity are commonly sustained in adolescent males during sport activities which involve jumping and tackling. Tibial tuberosity avulsions combined with lateral tibial plateau in an adolescent are rare injuries. We received an unusual case of left Ogden type IIIB avulsion fracture of the tibial tuberosity with an articular involvement of the lateral tibial plateau (Salter Harris type IV) in a 14-year-old boy. The injury was sustained after jumping from three stairs where the boy predominantly landed on his left lower limb. Unlike other cases reported, since they were athletes and injuries were sustained during sport activities, our case is an obese individual who jumped off few stairs. The results following surgical treatment are excellent since the fracture united, and the articular surface was restored without the need of arthrotomy with gaining full range of motion. Therefore, displaying this case in the literature will be helpful since it is rare and was successfully treated differently from the previously reported cases.

## 1. Introduction

An avulsion fracture of the tibial tuberosity is an uncommon injury, with reported incidence ranging from 0.4% to 2.7% (as cited in Bolesta MJ, 1986; Mosier SM, 2004) [1]. The average age of presentation of such injuries was reported to be 15.0 ± 1.1 years with around 10% of the cases initially presented with compartment syndrome or vascular compromise [2]. It accounts for less than 1% of all physeal injuries [2]. Avulsion of the tibial tubercle can occur when the traction force on the patellar ligament exceeds the combined strength of the physes underlying the tubercle, the surrounding perichondrium, and the adjacent periosteum [3]. There are two mechanisms of injury: violent contraction of the quadriceps muscle against a fixed tibia which can occur in forceful jumping or acute passive flexion of the knee against the contracted quadriceps [4]. Associated injuries may involve the surrounding ligaments, menisci, and rarely tibial plateau [5].

## 2. Case Report

**Figure 1:**
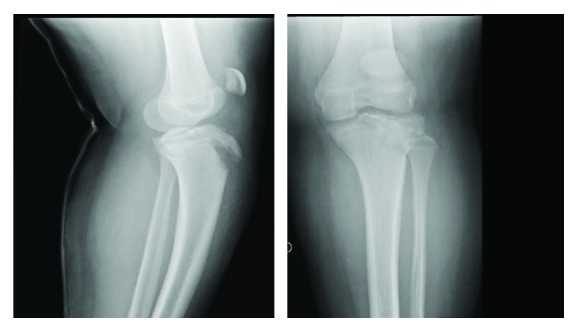
AP and lateral view X-ray of the left knee.

**Figure 2:**
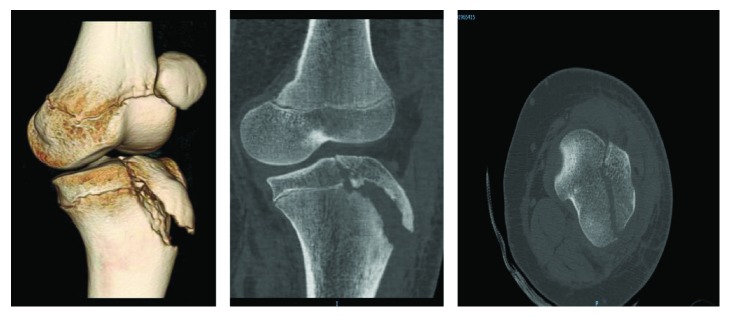
3D, sagittal, and coronal cuts of CT scan.

A medically free 14-year-old male who was obese sustained an injury to his left knee after jumping from 3 stairs. The patient mentioned that he predominantly landed on his left lower limb with his left knee in full extension and in external rotation. The patient started complaining of left knee pain limiting his range of motion and ability to bear weight immediately after the fall. He was brought to the Emergency Department (ER) of King Saud Medical City (KSMC) by his parents immediately after the injury. On physical examination, the left knee was profoundly swollen and bruised. There was tenderness over the tibial tuberosity and lateral joint line. He was unable to actively move the knee joint. The passive range of motion was painful. There were no signs indicating compartment syndrome or neurological or vascular injury. X-ray radiographs revealed a Watson-Jones type IIIB avulsion fracture of the tibial tuberosity apophysis (Figure 1). A CT scan showed a step of the articular surface more than 2 mm extending to the posterior-lateral epiphyseal part of the proximal tibia (lateral tibial plateau) (Figure 2).

**Figure 3:**
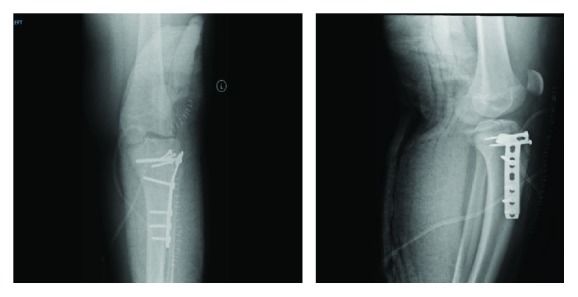
AP and lateral X-ray of the left knee postoperative day 1.

The patient was admitted and was prepared for operative management. A procedure was planned and done on a radiolucent table under general anesthesia. A tourniquet was used to avoid excessive bleeding during the procedure. The tourniquet was inflated after pulling down the quadriceps to avoid blocking the reduction due to the extensor mechanism. The leg is prepped and draped according to the standard orthopedic protocol. Anterolateral approach of the knee was used with an incision starting from the lateral upper border of the patella to 10 cm down. Deep fascia was opened anterior to the iliotibial tract. The fracture line was identified; the reduction of the articular step was done using a reduction clamp and assured using a portable image intensifier on flexion and extension of the knee without arthrotomy. We avoided arthrotomy of the joint to not make it vulnerable to infection and possible scarring. Stabilization of the reduction was maintained using a k-wire. Definitive fixation was achieved with three 3.5 mm partially threaded cancellous screws placed under fluoroscopic guidance for the tibial tuberosity fracture. A proximal tibial plate was slid laterally and was used to buttress the lateral tibial column. Careful placement of the screws was done to not cross the physis with the help of a C-arm (Figure 3). After fixation, good hemostasis was achieved, drain was placed, and the range of motion was assessed which was full. Closure was done layer by layer, then dressing after. The postoperative plan was to immobilize the knee in a cylindrical cast for 3 weeks with no weight bearing on the left lower limb with the use of crutches for ambulation.

**Figure 4:**
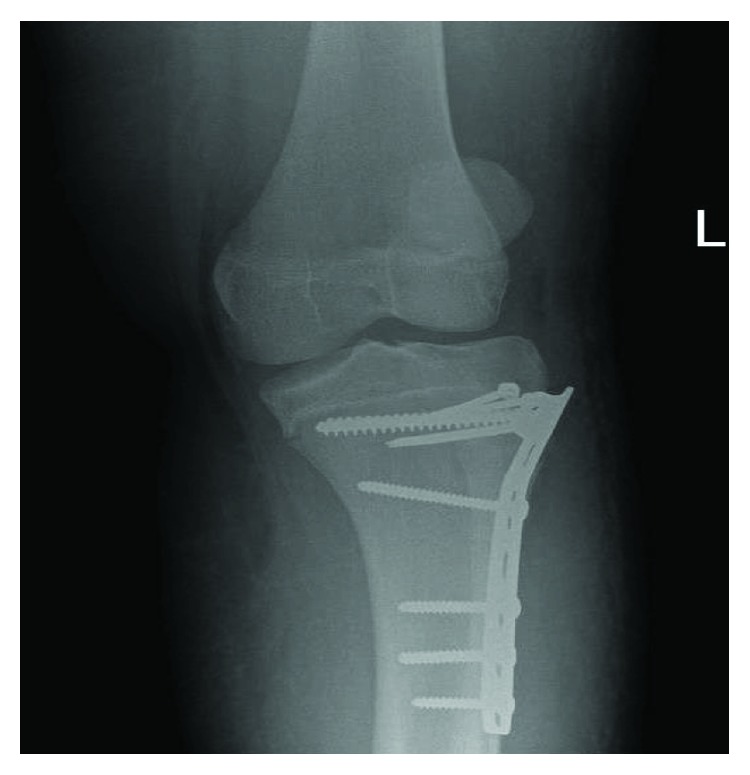
AP X-ray of the left knee 3 weeks post-op.

Postoperative knee CT scan is requested to ensure that the fracture is anatomically reduced. The patient received analgesia and antibiotics, and drain was removed 24 hours post-op. The patient was seen in an orthopedic clinic after 3 weeks, there were no signs of surgical site infection, and the clips were removed. The controlled range of motion was advised using a hinged knee brace throughout the day for 4 weeks. A follow-up X-ray (Figure 4) shows that the fracture is aligned with no loss of reduction or displacement. Physiotherapy is advised 7 weeks postoperative management focusing on the range of motion and strengthening. The patient was seen 6 weeks later, he had full range of motion with no deformity, and there were no complaints reported by the patient like locking or pain.

## 3. Discussion

The tibial tuberosity develops from a secondary ossification center in the proximal tibia between 7 and 9 years of age [2, 6]. During ossification, calumniated cartilaginous cells with poor tensile strength transiently replace the fibrocartilage, predisposing the tibial tuberosity to traction injury just before or during the later stages of physiologic epiphysiodesis [2, 6]. The mechanism of injury is usually an indirect force caused by sudden contraction of the quadriceps muscle. During sudden acceleration and deceleration forces, the quadriceps mechanism forcefully contracts against the patellar tendon insertion. When the force is greater than the strength of the tibial tubercle physis, a fracture occurs, leading to avulsion of the tibial tubercle [1, 2]. An important predisposing factor is a preexisting Osgood-Schlatter disease which was excluded and was not evident in our case [1]. Tibial tubercle avulsion fractures are commonly seen in athletic males, reportedly basketball players (Ozer H, 2002) [2]. The most common self-reported mechanism of injury was related to jumping activities followed by falling directly on the knee and twisting injuries [2]. Our patient was not athletic, he sustained an injury while jumping over three stairs landing on the left lower limb with the knee extended and externally rotated in relation to the femur which combines sudden quadriceps contraction and a twisting mechanism. Basketball usually involves frequent jumping which will lead to repetitive contractions and could be a factor leading to a tibial tubercle avulsion fracture. We believe that the weight of the patient was a very important factor since he was weighing 105 kilograms (BMI: around 40 kg/m^2^). Javed et al. [7] reported a Watson-Jones type III avulsion fracture of the tibial tuberosity apophysis and an Aitken type II fracture of the lateral tibial plateau which is similar to our case. Their definitive fixation was achieved with five 4.5 mm partially threaded cancellous screws which revealed a satisfactory outcome. Our case was surgically treated differently since reduction was achieved with three 3.5 mm partially threaded cancellous screws for the tibial tuberosity fracture and a proximal tibial plate was used to buttress the lateral tibial column. The full range of motion was gained 12 weeks postoperative management without symptoms as reported previously [7]. Our surgical management is similar to what Chakraverty et al. [8] reported in a case series of skeletally mature patients with tibial tubercle fractures in association with tibial plateau fractures. Prior to fixing the tibial plateau fracture, we used 3 cancellous screws to fix the tibial tuberosity anatomically since the growth plate was open. Howarth et al. [9] approached the reduction of tibial tubercle fractures with intra-articular involvement using small parapatellar arthrotomy for both inspections of associated meniscal or osteochondral injuries as well as subsequent confirmation of anatomic articular reduction with arthroscopic assistance. We avoided opening the joint capsule to decrease the chance of infection, arthrofibrosis, and complex regional pain syndrome [10]. Our case is discharged from our clinic without complications and with no residual complaint after 13 weeks.

## 4. Conclusion

The avulsion fracture of the tibial tuberosity combined with lateral tibial plateau is a rare fracture to be encountered, and few have been reported in the literature with similar treatment approach but not exactly the same. The mechanism of the injury is an important factor in the identification of the structures involved. Unlike the previously reported cases, the patient was not athletic and was obese. Open reduction of such case can be obtained without the need of arthrotomy.

## References

[1] Pretell-Mazzini J., Kelly D. M., Sawyer J. R. (2016). Outcomes and complications of tibial tubercle fractures in pediatric patients: a systematic review of the literature. *Journal of Pediatric Orthopedics*.

[2] Pandya N. K., Edmonds E. W., Roocroft J. H., Mubarak S. J. (2012). Tibial tubercle fractures: complications, classification, and the need for intra-articular assessment. *Journal of Pediatric Orthopedics*.

[3] Hamilton S. W., Gibson P. H. (2006). Simultaneous bilateral avulsion fractures of the tibial tuberosity in adolescence: a case report and review of over 50 years of literature. *The Knee*.

[4] Jakoi A., Freidl M., Old A., Javandel M., Tom J., Realyvasquez J. (2012). Tibial tubercle avulsion fractures in adolescent basketball players. *Orthopedics*.

[5] Frey S., Hosalkar H., Cameron D. B., Heath A., David Horn B., Ganley T. J. (2008). Tibial tuberosity fractures in adolescents. *Journal of Children's Orthopaedics*.

[6] Brey J. M., Conoley J., Canale S. T. (2012). Tibial tuberosity fractures in adolescents: is a posterior metaphyseal fracture component a predictor of complications?. *Journal of Pediatric Orthopedics*.

[7] Javed S., Barkatali B., Siddiqui M., Sarin R. (2013). Combined avulsion fracture of the tibial tuberosity and lateral tibial plateau in an adolescent: case report. *Malaysian Orthopeadic Journal*.

[8] Chakraverty J. K., Weaver M. J., Smith R. M., Vrahas M. S. (2009). Surgical management of tibial tubercle fractures in association with tibial plateau fractures fixed by direct wiring to a locking plate. *Journal of Orthopaedic Trauma*.

[9] Howarth W. R., Gottschalk H. P., Hosalkar H. S. (2011). Tibial tubercle fractures in children with intra-articular involvement: surgical tips for technical ease. *Journal of Children's Orthopaedics*.

[10] Mayr H. O., Stoehr A. (2016). Komplikationen arthroskopischer Eingriffe am Kniegelenk. *Der Orthopäde*.

